# A Switch in the Control of Growth of the Wing Imaginal Disks of *Manduca sexta*

**DOI:** 10.1371/journal.pone.0010723

**Published:** 2010-05-19

**Authors:** Alexandra Tobler, H. Frederik Nijhout

**Affiliations:** Department of Biology, Duke University, Durham, North Carolina, United States of America; Stockholm University, Sweden

## Abstract

**Background:**

Insulin and ecdysone are the key extrinsic regulators of growth for the wing imaginal disks of insects. *In vitro* tissue culture studies have shown that these two growth regulators act synergistically: either factor alone stimulates only limited growth, but together they stimulate disks to grow at a rate identical to that observed *in situ*. It is generally thought that insulin signaling links growth to nutrition, and that starvation stops growth because it inhibits insulin secretion. At the end of larval life feeding stops but the disks continue to grow, so at that time disk growth has become uncoupled from nutrition. We sought to determine at exactly what point in development this uncoupling occurs.

**Methodology:**

Growth and cell proliferation in the wing imaginal disks and hemolymph carbohydrate concentrations were measured at various stages in the last larval instar under experimental conditions of starvation, ligation, rescue, and hormone treatment.

**Principal Findings:**

Here we show that in the last larval instar of *M. sexta*, the uncoupling of nutrition and growth occurs as the larva passes the critical weight. Before this time, starvation causes a decline in hemolymph glucose and trehalose and a cessation of wing imaginal disks growth, which can be rescued by injections of trehalose. After the critical weight the trehalose response to starvation disappears, and the expression of insulin becomes decoupled from nutrition. After the critical weight the wing disks loose their sensitivity to repression by juvenile hormone, and factors from the abdomen, but not the brain, are required to drive continued growth.

**Conclusions:**

During the last larval instar imaginal disk growth becomes decoupled from somatic growth at the time that the endocrine events of metamorphosis are initiated. These regulatory changes ensure that disk growth continues uninterrupted when the nutritive and endocrine signals undergo the drastic changes associated with metamorphosis.

## Introduction

In insects, as in other organisms, nutrition is necessary for normal growth. *In vitro* studies have shown that nutrition does not act directly on cells but typically exerts its effect via hormonal signals such as insulin-like peptides and ecdysteroids [1], [2], [3]. When insect larvae enter metamorphosis feeding stops and somatic growth ceases, but the imaginal disks continue growing at their normal rate. Evidently growth of the imaginal disks becomes uncoupled from nutrition at some time during the last larval instar. In this paper we investigate the biological mechanism of this developmental switch in *Manduca sexta*.

Insulin-like peptides are the most common mediators between nutrition and growth [4], [5], [6]. Nutrition, via circulating sugar levels, promotes the release of insulin from neurosecretory cells in the brain into the hemolymph, and then acts on peripheral tissues to stimulate protein synthesis and cellular growth [5], [7]. Insulin-like peptides have been identified in a variety of insects [8], [9], [10] and their function has been best studied in *Drosophila melanogaster* [reviewed in 11]. In *Drosophila*, ablation of the insulin producing cells in the brain leads to reduced larval growth and small adult flies [12]. Mutations in the insulin receptor or the insulin receptor substrate likewise result in a reduction of body size [6]. Over-expression of the insulin-like peptides during larval development results in large but normally proportioned adult flies [13].

The insulin-like peptides of the Lepidoptera are brain neurosecretory hormones called bombyxins (Bbx). Bbx were first identified in the silkworm *Bombyx mori* [10] and since then have been identified in other Lepidoptera, including the tobacco hornworm *Manduca sexta* [2], [14]. Bbx has been shown to be an essential growth factor for wing imaginal disks in *Precis coenia* and *M. sexta* [2], [3]. However, *in vitro* tissue culture studies have shown that Bbx by itself stimulates only weak growth, and that normal growth also requires the action of the steroid molting hormone, ecdysone [2], [3].

Ecdysone is controlled by the brain via the neurosecretory prothoracicotropic hormone and is present throughout larval growth at a low concentration. At the end of the growth phase ecdysone rises and induces the cessation of feeding and entry into the prepupal wandering stage, and two days later a large peak of ecdysone induces the pupal molt [15], [16], [17], [18]. Ecdysone by itself cannot stimulate normal growth of the imaginal disks *in vitro*, and it appears that ecdysone acts synergistically with Bbx to promote normal growth in the wing imaginal disks [2], [3].

In addition to ecdysone and Bbx, the fat body also plays a significant role in growth regulation [19], [20]. The fat body is the insect's equivalent of the liver, and is the major tissue for metabolism and storage of nutrients, and the source of most of the proteins that circulate in the hemolymph [21]. The fat body appears to produce a factor that stimulates cell proliferation and growth [19], [20]. Although the brain and the fat body are both capable of acting as sensors for nutritional conditions, their individual contributions to growth regulation are poorly understood, as are the mechanisms by which the signals they produce might interact to coordinate growth.

In the present study we examine the roles of nutrition, Bbx, ecdysone, and the fat body in the regulation of wing imaginal disk growth in the final larval instar of the tobacco hornworm *M. sexta*. We show that the relationship between nutrition and growth changes dramatically as the larva passes the critical weight. After larvae pass the critical weight blood trehalose, disk growth, and the transcript levels of the Bbx and insulin receptor (InR) genes become independent of nutrition. As a result, imaginal disk growth becomes decoupled from somatic growth, and wing disks continue to grow when starvation, or entry into the wandering stage, stops somatic growth. In addition, after the larva has passed the critical weight, factors from the abdominal region are required to drive continued growth. Finally, we discuss the comparative physiology of the developmental transition that happen when larvae pass the critical weight, by comparing the distinctively different developmental strategies employed by *Manduca* and *Drosophila*.

## Results

### Normal growth of body and wing disks

**Figure 1:**
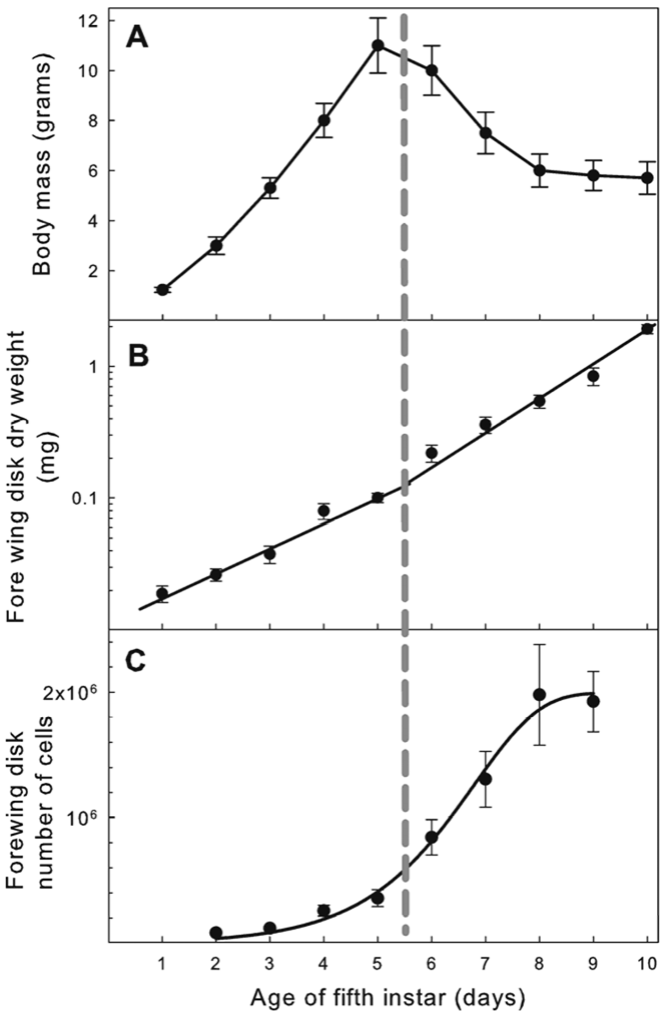
Somatic and wing imaginal disk growth during the last larval instar of *Manduca sexta*. Vertical dashed line between days 5–6 indicates the transition from the feeding to the wandering phase. (A) Body growth; feeding stops and mass declines during the wandering phase. Each point is the mean of >50 individuals; bars are standard deviations. (B) Semilogarithmic plot of growth in dry mass of the fore wing imaginal disks. The growth rate of the wing disk increases after larvae enter the wandering stage. Lines are exponential regressions. During the feeding phase the growth is given by the exponential equation mass = 0.011e^0.45^, r^2^ = 0.93; during the wandering phase growth is given by the exponential equation mass = 0.007e^0.55^, r^2^ = 0.97. Each point is the mean of 20–22 individuals; bars are standard deviations. (C) Semilogarithmic plot of the increase in cell number of the fore wing imaginal disk. Cell division stops after day 8. Each point represents the mean of 12 individuals; bars are standard deviations.

Under normal feeding conditions fifth instar larvae feed for 5 days and then enter the wandering phase, a non-feeding prepupal stage. Larval mass increased at an approximately exponential rate during the feeding phase, but after the larva entered the wandering phase, body mass decreased due to the purging of the gut contents (Figure 1A). The cessation of larval growth and entry into the prepupal stage is controlled by secretion of the steroid hormone, ecdysone [17], [22]. The wing imaginal disks, by contrast, continued to grow uninterrupted throughout both the feeding stage and non-feeding wandering stage. The wing disks increased in mass (dry weight) at an exponential rate (Figure 1B), and this rate increases slightly when larvae enter the wandering stage [3]. The cell number in the forewing disks increased exponentially through most of the feeding and wandering stage, with a doubling time of 30±3 h, but on day 8 of the 5^th^ instar cell division in the wing disk stops and growth ceases (Figure 1C). Cell division and growth resume again after pupation. The continued increase in dry weight after cell division has stopped (Figure 1B) is due to the fact that the cells of the disk begin to secrete a cuticle that will become the pupal wing integument. At the beginning of the 5^th^ larval instar, the forewing imaginal disks had approximately 80,000 cells, at the end of the feeding stage there were about 600,000 cells, and at the end of the wandering stage about 1.6 million cells.

### Influence of nutrition on wing growth

**Figure 2:**
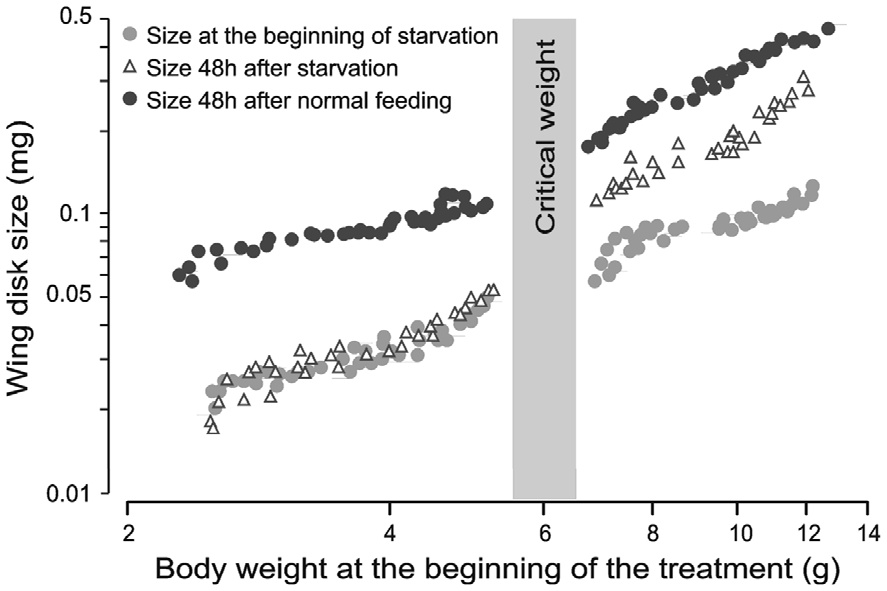
Effect of starvation before and after critical weight on wing imaginal disk growth. The grey bar represents the mass interval at which 95% of larvae attain the critical weight. Before the critical weight starvation (open triangles) stopped growth. After the critical weight starvation did not stop growth but reduced the growth rate to approximately 66% of control (black circles).

Food deprivation had a different effect on wing disk growth depending on when it occurs. When larvae were starved before they had attained their critical weight both body and wing disks stopped growing. By contrast, when larvae were starved after they had reached the critical weight the body stopped growing but the imaginal disks did not (Figure 2). Wing disks from larvae starved after the critical weight showed a significant increase in size in 48h (*p*<0.0001), although their growth rate was somewhat lower than that of disks from feeding larvae of the same age (*p*<0.0001) (Figure 2).

The critical weight marks a developmental transition. At the critical weight the secretion of juvenile hormone (JH) stops, and there is an increase in expression of JH-esterase, the primary catabolic enzyme for JH [15], [23], [24]. This change in JH metabolism sets in motion a time-invariant process that leads to the secretion of the small surge of ecdysone that terminates the feeding phase [22]. This time-invariant period is independent of nutrition, so the critical weight is operationally defined as the size at which further feeding is not necessary for a normal time course to pupation [25], [26], [27]. For our laboratory strain of *M. sexta* the critical weight is between 6.0–6.5g. The peak weight of this strain is 11–12g, so the critical weight occurs about halfway during the growth phase. Larvae that were starved after the critical weight reached the wandering and pupal stages at the same time as normally feeding control larvae, but developed into small adults with small but normally-proportioned wings. Larvae that were starved before critical weight had a significant delay of entry into the wandering stage and never emerged as adults.

### Effect of juvenile hormone on wing disk growth

**Figure 3:**
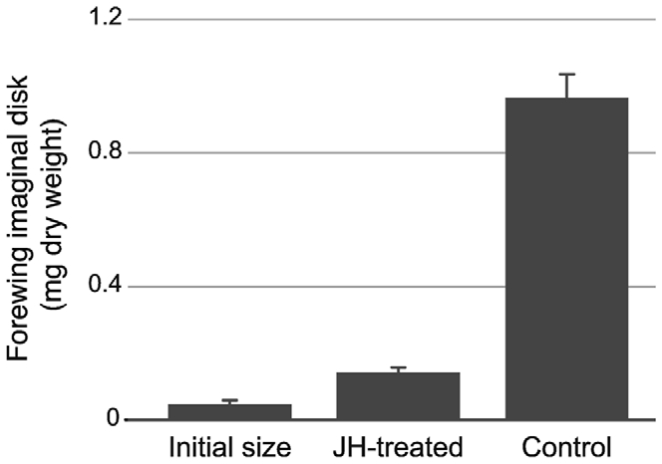
Effect of juvenile hormone on wing disk growth. Graph shows the size of the disks after 4 days for JH-treated larvae and acetone-treated controls. Each bar represents the mean of 6–8 larvae. Error bars SEM.

One of the effects of starvation of larvae before they have reached the critical weight is that the level of JH in the hemolymph goes up [28]. JH is known to inhibit the growth of imaginal disks in *Precis coenia* [29], so it is possible that the inhibition of imaginal disk growth during starvation in *M. sexta* was mediated by JH. We tested this possibility by measuring imaginal disk growth of JH-treated larvae. A 0.1µg dose in 10µl acetone of the JH-analog methoprene was topically applied on three successive days to feeding larvae, starting at weights between 9–10g, and disks were dissected 24h after the last treatment. JH-treated larvae grew normally, to large size, but their entry into wandering stage was inhibited by JH. Control larvae had entered the wandering stage at the end of the experiment. The forewing disks of JH-treated larvae grew moderately, compared to acetone-treated controls. They tripled in size during the experimental period (Figure 3), reaching a size approximately equal to that of disks of larvae on the day they enter the wandering prepupal stage. By contrast, the wing disk of acetone-treated controls increased in size almost 20-fold during the experimental period. Evidently elevated levels of JH are able to significantly restrict the growth of wing imaginal disks.

### Influence of brain and abdomen on wing disk growth

**Figure 4:**
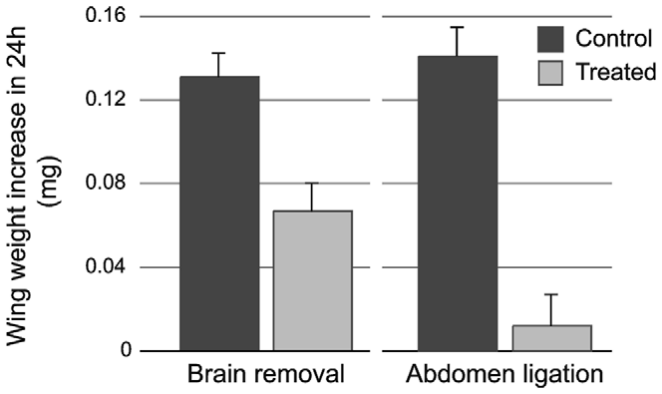
Effect of the brain removal and abdominal ligation on wing imaginal disk growth. Dry weight gain after 24h of brain removal (*p*<0.001) or abdominal ligation (*p*<0.001) for the forewing disk of first day wandering phase (day 6). Each bar represents the mean in forewing disk size of 18–20 disks. Error bars SEM.

**Figure 5:**
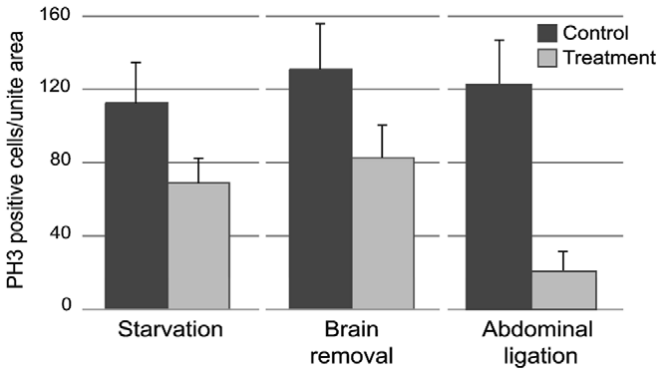
Quantification of cell proliferation in the forewing imaginal disk. Cell proliferation was measured as the average number of PH3 positive cells in an area of 0.0875mm^2^ of the wing imaginal disk. Effect of starvation: larvae were starved on day 4 of the feeding phase and PH3 cells in the wing disk were counted 48h later (*p* = 0.021). Effect of brain removal: the brain was removed the first day of the wandering phase (day 6) and PH3 cells were counted 24h later (*p*<0.001). Effect of abdominal ligation: a ligation was placed between the thorax and the abdomen on day 6 larvae, and PH3 cells were counted 24h later (*p*<0.001). Each bar represents the mean of 10 disks. Error bars SEM.

Growth factors in insects are produced by both the brain and the fat body [2], [5], [13], [19], [30]. The fat body is mostly located in the abdominal region surrounding the gut. We used brain extirpation and body ligation to test the interaction of factors coming from the brain and the abdominal region on disk growth. Both treatments were performed when the larvae had entered the wandering stage and, therefore, feeding had ceased. Brain extirpation did not cause wing disks to stop growing (Figure 4). However, the disks from brainless larvae had a significantly lower rate of cell proliferation than disks from sham-operated control larvae (*p*<0.001) (Figure 5). Brainless larvae continued to develop and eventually pupated, but never eclosed as adults.

A ligation applied between the third thoracic segment and the abdomen isolates wing disks from most of the signals coming from the abdomen and, therefore, the fat body. Wing disks from such abdomen-ligated larvae stopped growing soon after the ligation was placed. Wing disks dissected before ligation and at 24h after ligation were not significantly different in size (*p* = 0.39) (Figure 5). Wing disks from abdomen-ligated larvae showed significantly lower cell proliferation that those of unligated larvae (*p*<0.0001) (Figure 5).

**Figure 6:**
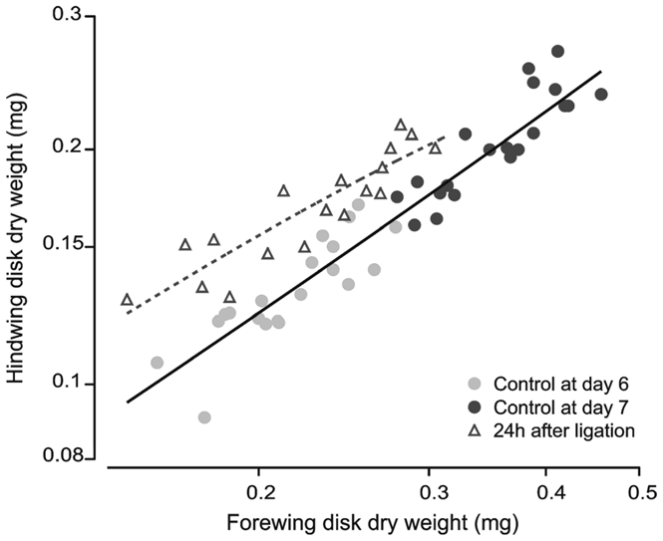
Effect of mid-thoracic ligation on fore- and hindwing imaginal disk growth. A ligation was placed between the second and third thoracic segment on the first day of the wandering phase (day 6). The ligation prevented the forewing from receiving most factors coming from the abdominal region, and also prevented the hindwing from receiving brain factors. The relationship between fore- and hindwing sizes for controls fall on a common regression. In ligated larvae (open triangles) the forewing disks grew little in 24 hours, and the hindwings grew about half as much as controls (black circles) The regression lines for control and ligated larvae for fore- vs hindwing disk sizes are statistically different (*p*<0.001).

Because fore- and hindwings are located on the second and third thoracic segments, respectively, a ligation placed between the second and third thoracic segments isolates the forewings from most factors coming from the abdomen, and simultaneously isolates the hindwings from factors produced by the brain. After a thorax-ligation the ratio between hind- and forewing mass increased significantly compared to that of non-ligated larvae (*p*<0.001) (Figure 6). Hindwings that had access to factors coming from the abdomen showed a significant increase in size after 24h, while forewings that had access to brain factors, but were deprived from abdominal factors, showed a reduced increase in size. This result suggests that factors from the abdomen are more effective at promoting growth than factors from the brain.

### Hemolymph sugar concentrations

**Figure 7:**
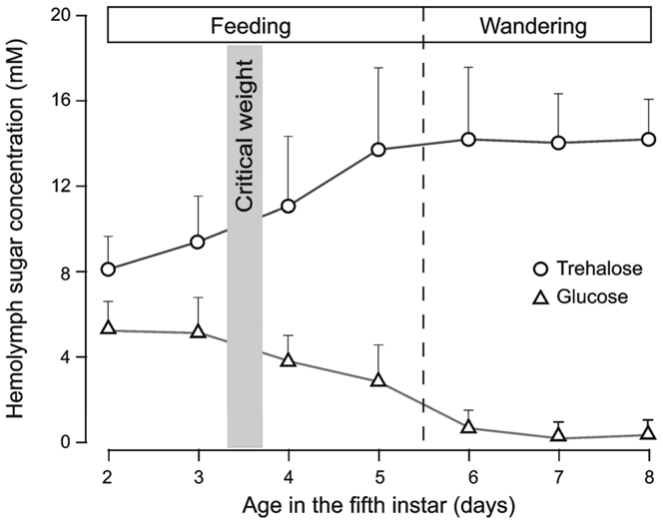
Hemolymph glucose and trehalose concentration during the last larval instar. Values are means of 10–16 individuals ±SD.

The primary source of growth promoting signals in the abdomen is the fat body. The insect fat body not only store reserves but also regulates the hemolymph composition of organic molecules, including carbohydrates. Growth of wing disks requires insulin signaling [2], [3], whose secretion is stimulated by carbohydrates [7]. In most insects, the primary hemolymph carbohydrate is trehalose, with glucose typically present as a minor component [31]. *M. sexta* hemolymph has both trehalose and glucose in significant concentrations. In feeding larvae, both glucose and trehalose concentration changed greatly with age in the final instar (Figure 7). Glucose levels started at about 5.5mM, and gradually declined to undetectable levels on the day when the larva entered the wandering phase. Trehalose levels were about 8mM at the beginning of the instar, and gradually increased to about 14mM when larvae entered the wandering phase, and were maintained at that level during the rest of the wandering phase (Figure 7).

### Effect of starvation on hemolymph sugar concentration

**Figure 8:**
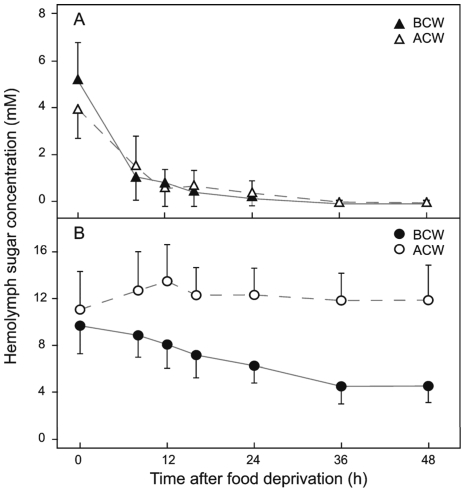
Effect of starvation on hemolymph glucose and trehalose concentration. (A) Glucose concentration in larvae starved before (BCW) and after critical weight (ACW). (B) Trehalose concentration in larvae starved before and after critical weight. Values are means of 8–16 independent samples ±SD.

Larvae were starved either before or after passing their critical weight and their hemolymph glucose and trehalose concentrations were measured. Nutrient deprivation affected glucose and trehalose concentrations differently, and the effect also depended on the timing of the starvation. Glucose levels declined precipitously soon after the animal had been deprived of food. Eight hours after food deprivation glucose concentration had fallen from about 5.5mM to 0.5mM, and 24h later, glucose in the hemolymph had become almost undetectable. This response was the same regardless of whether starvation occurred before or after the larva had reached the critical weight (Figure 8A). By contrast, the response of trehalose to starvation differed dramatically depending on whether the larva had attained critical weight or not. In larvae starved before the attainment of critical weight, the trehalose level declined gradually during the first 24h and reached a minimum of 4.5mM at 36h after starvation. However, when larvae were starved after reaching critical weight, the trehalose concentration rose slightly until 12h after the beginning of starvation, after which it remained nearly constant at around 12mM (Figure 8B).

### Effect of glucose and trehalose injection

**Figure 9:**
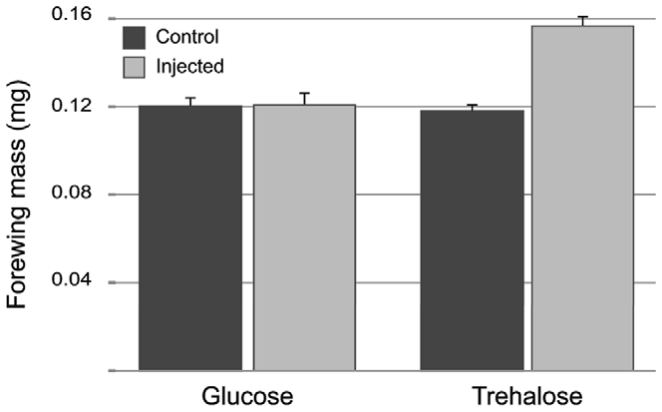
Effect of glucose and trehalose injection on wing disk growth. Glucose and trehalose were injected 3 times at 12h interval during 48 h. Each bar represents the mean of 6–10 disks. Error bars SEM.

To investigate whether the variation in carbohydrate concentrations was functional, we injected 5^th^ instar larvae that were starved before they reached the critical weight with either glucose (1mg) or trehalose (0.5mg) before they had passed the critical weight. The carbohydrate was injected 4 times at 12h intervals, starting 6h after starvation, and the forewing imaginal disks were dissected out at 4h after the last injection. Glucose injections did not stimulate wing disk growth; by contrast, trehalose injections stimulated significant wing disk growth (Figure 9). We suspect that glucose had little or no effect on disk growth because it is very rapidly cleared from the hemolymph after injection (half-life of injected glucose in hemolymph was <1h), whereas trehalose levels persisted for a longer time and could provide for the slow release of glucose. The wing disks of trehalose-injected larvae did not grow as much as the disks of feeding larvae, possibly because the pattern of injections was not able to establish the normal level of trehalose. We also injected glucose and trehalose in ligated larvae. Wandering stage larvae were ligated between the meso- and metahorax and 24h later glucose (1mg) or trehalose (0.5mg) was injected into the anterior and posterior compartments. After 24h of ligation the concentration of glucose in the anterior and posterior compartment of the ligation remained at undetectable levels. In contrast the concentration of trehalose after the ligation decreased in the anterior compartment (9.2mM) and remained high in the posterior compartment (13.3mM). Injection of glucose had no effect on wing disk growth in either compartment. Trehalose stimulated a small amount of disk growth in the anterior compartment but no growth in the posterior compartment. These results stand in contrast to the findings of Masumura *et al.* [7] who showed that in *Bombyx mori* glucose, but not trehalose, stimulated the release of Bbx. This suggests that there may be species-specific differences in how carbohydrates are used to signal nutritional status in regard to imaginal disk growth. Indeed, it is commonplace to find significant species-specific differences in metabolic and developmental physiology among the insects [32], because many of these higher-level processes in post-embryonic development are involved with adaptations to particular life histories and environments.

### Effect of nutrition on bombyxin and insulin-receptor transcript levels

**Figure 10:**
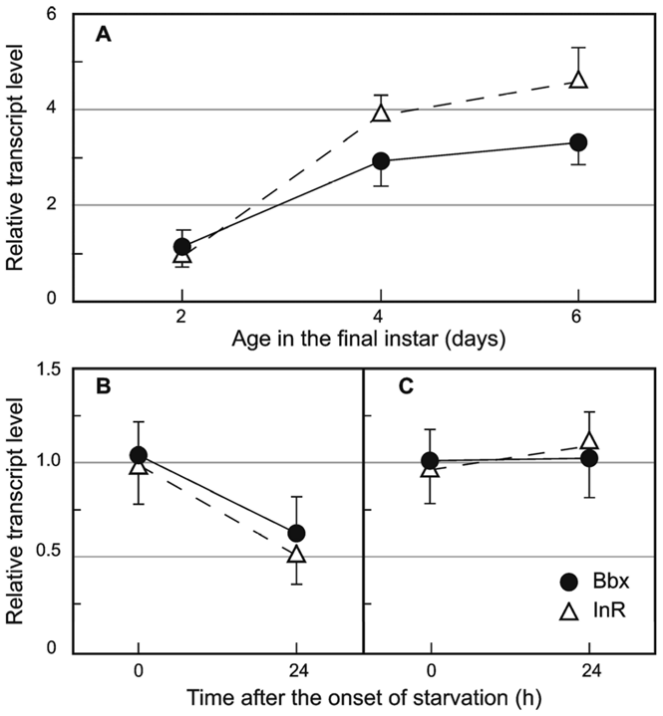
Bbx and InR mRNA transcript levels. (A) Last larval instar. Effect of food deprivation at day 2 (B), and at day 4 (C) of the feeding phase. Transcript levels were measured 24h after starvation. Each point represents the mean of 3–5 biological replicates measured in triplicate. Error bars SD.

To investigate whether the effect of nutrition was mediated via the insulin-signaling pathway we analyzed the expression of the neurosecretory insulin-like peptide Bbx in the brain, and of the insulin-receptor (InR) in the wing imaginal disks of normally-feeding 5^th^ instar larvae. The transcript level of both Bbx and InR increased gradually in the course of the instar (Figure 10). The effect of food deprivation on Bbx and InR transcript levels depended on the time of larval development (Figure 10). In larvae starved before the critical weight Bbx and InR mRNA levels declined dramatically compared to those of feeding larvae (Bbx: *p* = 0.0022, InR: *p* = 0.0083). However, when larvae were starved after having attained the critical weight, neither Bbx nor InR mRNA transcript levels changed relative to those of feeding controls (*p*>0.05) (Figure 10).

## Discussion

### The shifting endocrine environment at the end of larval life

At the end of larval life the internal environment of insects undergoes dramatic changes as the endocrine and physiological processes that culminate in metamorphosis begin to unfold. During this period there is also a major shift in the regulation of growth, associated with major shifts in the intake of nutrients and in the hormones that act as extrinsic regulators of tissue growth. In the Lepidoptera three hormones control the growth of imaginal disks during this period: the insulin-like hormone bombyxin (Bbx), ecdysone, and juvenile hormone (JH).

*In vitro* culture studies have shown that normal growth of the wing imaginal disks of Lepidoptera requires Bbx, as well as low levels of ecdysone [2], [3]. Ecdysone needs to be above a threshold concentration of 0.05 to 0.1µg/ml [3], which is at or below the levels at which ecdysone normally fluctuates during the feeding and wandering phases, and substantially below the levels required to induce molting [15], [18].

During the last larval instar there is a complex and shifting interaction between these hormones and food intake. If larvae are starved at any time during the feeding phase, body growth stops. In contrast, the response of wing imaginal disks to starvation depends on the developmental stage of the larvae. In larvae that have not yet reached the critical weight, starvation causes an arrest in wing disk growth (see also [33]). But in larvae that have passed the critical weight, wing disk growth continues albeit at a slightly decreased rate (Figure 2). Thus after a larva passes the critical weight, growth of the wing imaginal disks becomes uncoupled from food intake. Several molecular events are associated with this transition. Prior to the critical weight the transcript levels of Bbx in the brain and the InR in the wing disks declines when larvae are starved, but after the critical weight these transcript levels are unchanged after starvation. Although the level of transcription does not necessarily correspond to the level of activity of the protein product, these finding indicate that the nutritive control of the signaling mechanisms for the regulation of imaginal disk growth changes after the larva passes the critical weight. What could be the cause of this switch in the mechanism that regulates imaginal disk growth?

### A role for juvenile hormone

One possible player in this switch is JH. Food deprivation of *M. sexta* early in the last instar causes the JH titers to rise [28], [34]. Exogenous JH can inhibit wing disk growth (Figure 3 and [29]), as can the rise in JH titer caused by starvation [28], [33]. Elevated JH titers could, therefore, explain the arrest in wing disk growth observed during starvation before the critical weight. We believe this is a mechanism to limit the otherwise explosive exponential growth of the imaginal disk under conditions of inadequate nutrition and slow somatic growth. After larvae pass the critical weight the secretion of JH stops and the rate of its catabolism goes up [15], [23], [27]. The absence of JH after the critical weight could therefore explain why disks continue to grow in larvae starved after the critical weight. But it does not explain why the rate of imaginal disk growth declines somewhat (Figure 2) in those starved larvae. Although Bbx is required for normal wing disk growth after the critical weight [3], our findings suggest that Bbx is not involved in modulating the growth rate during this time period to match disk growth to somatic growth. The slight decrease in the wing disk growth rate under starvation may be a direct effect of food deprivation.

### Abdominal factors

Although neither starvation nor removal of the brain after the critical weight inhibited the growth of wing disks, an abdominal ligation that isolated the wing disks from factors coming from the abdomen was effective at inhibiting growth. Apparently factors from the abdomen are necessary for wing disk growth. To verify this we placed thoracic ligatures so that the forewings were in contact with factors from the brain and the hindwings with factors from the abdomen. In such ligated larvae the forewings grew less than hindwings, which supports the idea that the abdomen produces factors that are necessary for wing disks growth.

*In vitro* cultures of wing imaginal disks have shown that fat body extracts can provide factors that are necessary for growth and development. In Diptera and Lepidoptera wing disk growth proceeded normally only when fat body extracts were added to the culture media [35], [36], [37]. Studies with *Drosophila* have demonstrated the importance of the fat body in the coordination of nutrition and growth, indicating a primary role for the fat body in the control of growth [19], [20], [38]. It seems likely, therefore that the abdominal factor required for imaginal disk growth after a larva passes the critical weight is produced by the fat body, although the possibility that neurosecretion from the ventral never cord plays a role cannot be excluded. The possibility that this factor is a fat body-specific Bbx cannot be excluded, although we have been unable to detect expression of Bbx in fat body during the larval development.

Another possibility is that the fat body factor is the disaccharide trehalose. Our experiments show that trehalose is a requisite stimulus for disk growth. Prior to the critical weight starvation leads to a rapid decline in the level of trehalose in the hemolymph, but after the critical weight starvation actually leads to a slight rise in trehalose. The mechanism by which trehalose stimulates growth is not clear. One possibility is that trehalose stimulates Bbx synthesis or release, although such a function has never been demonstrated. More likely is the possibility that trehalose is broken down into glucose, which stimulates Bbx release. Because glucose is rapidly cleared from the hemolymph it is not seen to accumulate.

### A reinterpretation of the comparative biology of nutrition and disk growth control in the last larval instar

We have shown that in *Manduca* the critical weight in the last larval instar marks a transition in the regulation of the growth of wing imaginal disks. An interesting parallel phenomenon has been described in *Drosophila* where there is also a point in the last larval instar after which wing imaginal disk growth becomes independent of nutrition [39]. This point is the minimal viable size [40]. If larvae are starved before the minimum viable size the growth of imaginal disks stops and larvae eventually die. But if starved above the minimum viable size imaginal disk growth continues and larvae pupate normally, albeit at a much reduced body size. The minimal viable size in *Drosophila* is also commonly called the critical weight [41], [42], [43], but, although the response of wing growth to starvation appears to be superficially similar to that we describe here for *Manduca*, the minimum viable size of *Drosophila* and the critical weight of *Manduca* are quite different developmental-physiological phenomena. In *Manduca*, the critical weight is part of the mechanism that controls body size at metamorphosis [25], [26], [44], [45], [46]. At the critical weight the JH titer in the hemolymph declines and the activity of JH-esterase increases. The disappearance of JH disihibits ecdysone secretion, which induces initiation of the wandering stage and subsequent pupation [47], [48], [49]. The terminal growth period (between the critical weight and the wandering stage) is identical for starved and feeding larvae [23], [25], [27], [50]. The terminal growth period can be experimentally extended in a dose-dependent way with exogenous JH, and shortened by removal of the corpora allata, the glands that secrete JH [48], [51]. Thus the time required to clear JH controls the duration of the terminal growth period.

The minimum viable size in *Drosophila*, by contrast, is not associated with a developmental event that can be shown to be independent of starvation, and there is no evidence for an inhibitory role of JH. Most importantly, the terminal growth period is not the same for feeding and starved larvae, but is shorter in larvae that are starved [15], [52], [53]. This shortening of the terminal growth period suggests that the act of starvation may actually be a trigger for the metamorphic molt. This suggestion is reinforced by the effect of a low-nutrient diet, instead of outright starvation. When larvae are fed a low-nutrient diet they grow more slowly and metamorphosis is delayed until they have reached nearly normal final size [54]. This indicates the operation of a mechanism for body size regulation in feeding larvae that delays metamorphosis until larvae have grown to their normal size. In *Manduca* this is the function of the critical weight.

Thus *Drosophila* responds very differently to starvation and to a low nutrient diet: under starvation metamorphosis is accelerated, but under low nutrition it is delayed. This response to starvation is similar to that described in *Onthophagus*, where starvation is known to trigger metamorphosis [55]. This may be an adaptation for survival on an evanescent food resource (e.g. a piece of rotting fruit). We'll call it the “bail-out” response: a developmental adaptation in species whose larvae are unlikely to be able to move to a new food source when the first one runs out. Even a low amount of nutrient inhibits the bail-out response, and allows larvae grow (slowly) until they reach their species-characteristic size (or until the food runs out). The bail-out response explains why in *Drosophila* the growth of internal organs becomes independent of nutrition when larvae are starved above the minimum viable weight: because starvation sets in motion the events that lead to pupariation, and those are nutrition-independent. The finding that ecdysone signaling is involved in mediating the switch in the developmental response to starvation that occurs at the minimal viable weight [41], [42] suggests the possibility that in *Drosophila* starvation induces metamorphosis by enhancing ecdysone secretion.

### Conclusion

In *Manduca* nutrition is an absolute requirement for disk growth before the critical weight, and the brain is in control of imaginal disk growth via the secretion of Bbx and the tropic regulation of ecdysone secretion. After a larva passes the critical weight growth of the wing disks becomes uncoupled from nutrition, and the inhibitory effect of JH on wing disk growth is abolished. After this point the brain is no longer required for the control of disk growth, and this role appears to be passed to factors originating from the abdominal region. The uncoupling of imaginal disk growth from nutrition and from the brain ensures that growth continues uninterrupted when the nutritive and cerebral neuroendocrine signals undergo the drastic changes associated with metamorphosis.

## Materials and Methods

### Experimental animals

Larvae of the tobacco hornworm *Manduca sexta* were reared on a standard laboratory diet in individual cups at 26°C under long-day conditions (16h light: 8h dark). Age during the fifth (last) larval instar was measured as time since ecdysis: the day of ecdysis to the last larval instar was designated as day 1 of the feeding phase. On day 6 most of the larvae had transitioned from the feeding phase to the wandering phase. The wandering phase lasted from day 6 to day 10, after which most animals pupated. All experiments were performed 2–4h after lights-on.

### Wing size measurement

Wing disks were dissected out after anesthetizing the larvae for 5 min in CO_2_. Wing disk dry weight was determined by rinsing dissected wing disks in water, placing them on a small tared disc of aluminum foil, and drying them at 60°C for 48h. Wing disk weight was determined to the nearest 1µg on a Cahn-25 Electrobalance. Cell number estimates were obtained by dissociating wing disks in 0.35M citric acid and counting the cells in a hemocytometer. Cells were counted in duplicate samples in a total volume of 0.1µl.

### Cell proliferation

To obtain an estimate of wing disk cell proliferation the number of cells in M phase were identified with a 1∶2000 dilution of anti-phosphorylated histone H3 antibody (PH3), conjugated to Alexa Flour® 488 (Cell Signaling Technology, Inc. # 9708). Wing disks were fixed for 1h in 3.7% formaldehyde in phophate-buffered saline (PBS; 130mM NaCl, 7mM Na_2_HPO_4_, 3mM NaH_2_HPO_4_, pH 7.2) for 1h at room temperature, followed by rinses in PBS and 1% Triton-X.

### Starvation

Fifth instar larvae were starved at different body weights (before/after attainment of critical weight) during the feeding phase and kept without food for 48 h. To prevent larvae from desiccating, their containers were sprayed with water twice a day.

### Brain removal

Removal of the brain was performed on CO_2_ anesthetized larvae through a small triangular incision in the front of the head capsule on the first day of the wandering phase (day 6). The brain was gently drawn out of the incision and removed with micro-scissors. The incision was sealed by applying a small drop of wax around the triangular flap of cuticle. The effect of the treatment was measured by comparing the difference in wing disk growth between brainless and sham-operated larvae after 24h.

### Ligations

Ligations were performed by placing a tight cotton thread around the larval body. Abdominal ligations were placed between the thorax and the abdomen. Thoracic ligations were placed between the second and third thoracic segment. The effect of this treatment was measured by comparing the differences in wing disk dry weight between ligated and non-ligated larvae after 24h.

### Hemolymph glucose and trehalose concentration

Glucose and trehalose (a disaccharide of glucose) are the two major forms of carbohydrates circulating *M. sexta* hemolymph. Hemolymph was collected in micro-centrifuge tubes by making an incision in the proleg. The concentration of glucose was determined by using the FreeStyle® Blood Glucose Monitoring System (Abbott). The limit of detectablilty for glucose is 0.2 mg/ml, so any concentration below was recorded as 0. Trehalose concentration was determined by incubating 100µl of hemolymph with 0.01 units of trehalase (Sigma, T8778) overnight at 37°C and measuring the amount of glucose liberated.

### RNA extraction

Total RNA was extracted using the RNeasy Mini Kit (Qiagen). Tissues were dissected in cold sterile insect saline. For a given determination the RNA was extracted from 6 brains or 3 wing disks.

### Expression of Bbx and InR

Transcript levels of bombyxin (Bbx) levels in brain tissue and InR levels in the wing disks were determined using real-time quantitative PCR (qPCR). First stranded cDNA was generated using oligo-dT priming with the Superscript® II kit (Invitrogen). Q-PCR was performed using an iCycler (Bio-Rad) with iQ™ SYBR® Green Supermix (Bio-Rad). Q-PCR reactions were performed according to manufactures instructions. Primers were designed based on cDNA sequences for *M. sexta* retrieved from genebank: Bbx (DQ080209), InR (FJ169464) and actin (L13764). Primers are: Bbx-Fw, AGTGCGCAGTGGTGTTGTGT and Bbx-Rv, ATAGTTCGTCCAGCGTGCAG; InR-Fw, GGGATTTCGGCATGACCAGAGATATT and InR-Rv, TCGTTCGACAGGCCCTGATATGG; Act-Fw, AAGGACCTGTACGCCAACAC and Act-Rv, ACATCTGCTGGAAGGTGGAC. Relative expression levels of Bbx and InR were calculated based on the ΔΔCt method [56] and normalized with actin levels. Each sample was run in triplicate and 3–5 biological samples were run for each of the experimental conditions.

### Statistical analysis

The effect of starvation on wing disk growth was analyzed using an analysis of covariance (ANCOVA) with body size at the beginning of the treatment as a covariate. The effect of brain removal and abdomen ligations on wing disk growth was analyzed using a one-tailed T-test. Differences in cell proliferation were examined using a mixed model nested analysis of variance (ANOVA). The effect of thoracic ligations on fore- and hindwing growth was examined by ANCOVA with forewing as a covariate. Transcript levels for Bbx and InR were analyzed using a nested two-tailed t-test. All statistical analyses were conducted using JMP® 7.0.2 (SAS Institute Inc., Cary, NC).
